# Medizinische Versorgung von Asylsuchenden in Erstaufnahmeeinrichtungen: Eine qualitative Bestandsaufnahme aus Versorgungsperspektive

**DOI:** 10.1007/s00103-020-03243-3

**Published:** 2020-11-12

**Authors:** Katharina Wahedi, Louise Biddle, Rosa Jahn, Sandra Ziegler, Steffen Kratochwill, Susanne Pruskil, Stefan Noest, Kayvan Bozorgmehr

**Affiliations:** 1 Sektion Health Equity Studies & Migration, Abteilung Allgemeinmedizin und Versorgungsforschung, Universitätsklinikum Heidelberg, Heidelberg, Deutschland; 2 Gesundheitsamt Altona, Hansestadt Hamburg, Hamburg, Deutschland; 3 Angewandte Gesundheits- und Pflegewissenschaften, Duale Hochschule Baden-Württemberg, Stuttgart, Deutschland; 4 Bevölkerungsmedizin und Versorgungsforschung, Fakultät für Gesundheitswissenschaften, Universität Bielefeld, Postfach 10 01 31, 33501 Bielefeld, Deutschland

**Keywords:** Asylsuchende, Medizinische Versorgung, Organisationsbezogene Versorgungsforschung, Ambulante Versorgung, Aufnahmeeinrichtung, Asylum seekers, Medical care, Organisational research, Ambulatory care, Reception centres

## Abstract

Im Zuge der gestiegenen Zahlen Asylsuchender in den Jahren 2015/2016 haben sich in Deutschland, geprägt durch lokale Akteur*innen, sehr unterschiedliche Konzepte zur medizinischen Versorgung in Aufnahmeeinrichtungen (AE) etabliert. Ziel unserer Studie war es, unterschiedliche Versorgungskonzepte in AE abzubilden und die Herausforderungen der Verstetigung bedarfsgerechter medizinischer Versorgungsstrukturen herauszuarbeiten.

Daten wurden aus 13 semistrukturierten Interviews und im Rahmen einer Fachtagung mit Workshops und Gruppendiskussionen erhoben und durch eine qualitative Inhaltsanalyse ausgewertet. Teilnehmer*innen waren Akteur*innen der medizinischen Versorgung in AE, darunter ärztliches und Gesundheitsfachpersonal, Verwaltungsbeauftragte, Vertreter*innen des öffentlichen Gesundheitsdienstes und Wissenschaftler*innen.

Als Antwort auf die gesundheitlichen Bedarfe von Asylsuchenden und die komplexen Rahmenbedingungen der Versorgung haben sich unterschiedliche Ambulanzkonzepte gebildet, deren Zweck, Organisation und Management in vielen Aspekten über das Angebot einer ärztlichen Sprechstunde hinausgehen. Die Ambulanzen unterschieden sich in organisationsbezogenen Aspekten z. B. hinsichtlich des Betreibers, der Personalstruktur und des Umfangs der Versorgung. Gemeinsame Herausforderungen stellen eine adäquate Bedarfsplanung, der Mangel einheitlicher Leitlinien und fehlende Schnittstellen zwischen den in der Ambulanz tätigen Akteur*innen dar. Dringender Handlungsbedarf im Sinne eines strukturierten und kontinuierlichen Erfahrungsaustauschs sowie in der Implementierung bundesweiter Standards ist geboten, um Ad-hoc-Initiativen in resiliente Ambulanzstrukturen zu überführen. Die erarbeiteten Handlungsbedarfe und Lösungsvorschläge können hierfür als Grundlage dienen.

## Abstract (en)

During the time of increased in-migration of asylum seekers to Germany in 2015 and 2016, different models of healthcare provision were established in reception centres, often on an ad hoc basis and influenced by local actors. The goal of this study was to map different care models and identify challenges in the implementation of effective and needs-based health service structures.

Data was generated through 13 semi-structured interviews and in an interactive workshop with group discussions. An analysis was conducted using a qualitative content analysis method. Participants were stakeholders from clinics in reception centres, including medical and healthcare personnel, administrators, representatives of public health offices and researchers.

Different models of ambulatory care have formed as a response to the particular medical needs of asylum seekers and the complex context in which care takes place, often exceeding the simple offer of primary care. The facilities fundamentally differ with regard to objectives and organisational aspects, e.g. the responsible carrier, structure of human resources and the extent of health services provided. Shared challenges include planning needs-based care, the lack of shared guidelines and a lack of opportunities for exchange between the different actors working in the clinics. Action is required to transform ad hoc initiatives into resilient health care practices, particularly regarding structured and continued opportunities for exchange, as well as the development and implementation of nation-wide guidelines. Jointly developed areas for action and proposed solutions presented here can serve as a basis for further work in this area.

## Einleitung

Um in Deutschland einen Antrag auf Asyl bei den Außenstellen des Bundesamtes für Migration und Flüchtlinge stellen zu können, müssen Asylsuchende in den ihnen zugewiesenen Aufnahmeeinrichtungen verbleiben (§22 Asylgesetz, AsylG). Aufnahmeeinrichtungen, je nach Bundesland auch als „Erstaufnahmeeinrichtungen“ oder „Landeserstaufnahmeeinrichtungen“ bezeichnet, sind daher sowohl erste Anlaufstelle als auch temporäre Unterkunft für Asylsuchende. Da im Rahmen der kurzfristig stark gestiegenen Zahlen neu ankommender Asylsuchender in den Jahren 2015 und 2016 die Kapazitäten der vorhandenen Aufnahmeeinrichtungen (AE) bei Weitem nicht ausreichten, wurden an vielen Standorten „ad hoc“ neue Unterkünfte aufgebaut [1–3].

Während des Aufenthalts in den AE sind die jeweiligen Landesbehörden zuständig für die Bedarfe der Asylsuchenden, darunter fällt auch die Sicherstellung der medizinischen Versorgung. Deren Umfang wird durch §4 des „Asylbewerberleistungsgesetzes“ (AsylbLG) bestimmt: Bis auf Einzelfallentscheidungen (§6 AsylbLG) ist die medizinische Versorgung auf die Behandlung akuter Erkrankungen und Schmerzzustände beschränkt. Die Versorgung der in den AE untergebrachten Asylsuchenden durch die Regelversorgung sicherzustellen, erweist sich als sehr herausfordernd. Zum einen erschweren verschiedene Barrieren, etwa eine mangelnde Kenntnis der Sprache und des deutschen Gesundheitssystems, den Asylsuchenden faktisch den Zugang. Zum anderen haben die geografisch nahe der AE gelegenen Praxen und Krankenhäuser häufig nicht die Kapazitäten, die Gesundheitsversorgung der Bewohner*innen zusätzlich zu übernehmen [4]. Daher wurden an vielen Standorten ärztliche Sprechstunden auf dem Gelände der AE eingerichtet [5]. Häufig entstanden solche Angebote aus dem akuten Bedarf heraus und sind daher von lokalen Akteur*innen und dem spezifischen Kontext der AE geprägt. Mangels bundes- oder landesweiter Vorgaben oder verbindlicher Standards haben sich inzwischen sehr unterschiedliche Versorgungsmodelle etabliert, über die vereinzelt in Form von „Gute-Praxis“-Modellen berichtet wurde, etwa in Hamburg [6, 7], Dresden [8] und Heidelberg [4], oder es wurden deskriptive Übersichtsarbeiten darüber veröffentlicht [9].

Die unterschiedlichen Versorgungsmodelle können einen wichtigen Einfluss auf die Qualität und den Zugang für Asylsuchende haben [5, 10]. Bereits eine im Sommer 2015 durchgeführte Studie zeigte, dass lokale Lösungen zwar situationsbedingt und temporär wertvolle Beiträge zur Versorgungssituation leisten können, jedoch für eine nachhaltige, bedarfsgerechte und evidenzbasierte Versorgung die Erarbeitung bundesweiter Standards geboten wäre [11]. Im Rahmen des vom Bundesministerium für Bildung und Forschung (BMBF) geförderten Forschungsvorhabens „RESPOND“ („Improving regional health system responses to the challenges of migration through tailored interventions for asylum-seekers and refugees“; www.respond-study.org) führten wir daher eine Studie mit den folgenden Zielen durch:

1. Qualitative Bestandsaufnahme der medizinischen Versorgung in verschiedenen Aufnahmeeinrichtungen in Deutschland, insbesondere der Entwicklung seit 2015,
2. Identifizierung von Barrieren und Herausforderungen zur Etablierung und Verstetigung nachhaltiger und bedarfsgerechter Versorgungsstrukturen,
3. Aufzeigen von Lösungsstrategien, Handlungsbedarf und Gute-Praxis-Beispielen zur Gewährleistung zugänglicher, bedarfsorientierter und qualitativ hochwertiger Versorgungsangebote.

## Methodik

**Figure 1:**
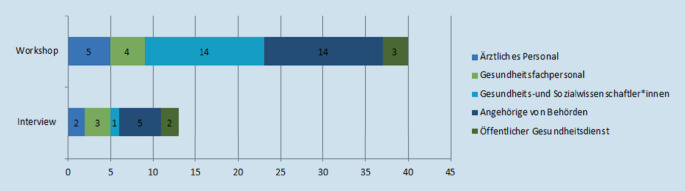

Die Datenerhebung bestand aus (1) einer qualitativen Interviewstudie und (2) einer eintägigen Fachtagung mit Workshops, Impulsvorträgen der Teilnehmer*innen, thematisch gebündelten Kleingruppendiskussionen und einem abschließenden Podiumsgespräch. Die Teilnehmer*innen der Interviewstudie und der Fachtagung waren Akteur*innen im direkten (Versorgung) und indirekten (Verwaltung/Organisationen) Umfeld medizinischer Ambulanzen in AE in Deutschland (Abb. 1).

Die qualitative Interviewstudie wurde im Zeitraum Dezember 2018 bis April 2019 durchgeführt. Basierend auf bestehenden wissenschaftlichen Erkenntnissen [11] sowie eigenen praktischen Erfahrungen in der Leitung medizinischer Ambulanzen [4] wurde ein semistrukturierter Interviewleitfaden erarbeitet. Die Interviewpartner*innen wurden anhand von Purposive Sampling (gezielte Auswahl der Interviewpartner) über bestehende Kooperationen und Kontakte ausgewählt, weitere Teilnehmer*innen wurden in einer Schneeballauswahl rekrutiert. Das Sample variierte hinsichtlich des Bundeslandes, ländlicher/städtischer Lage, beruflichen Hintergrunds und Funktion (ärztlich/Gesundheitsfachberufe/Verwaltung) und Betreiber der Ambulanz (öffentlicher Gesundheitsdienst/Universitätsklinik/privater Betreiber). Die Kontaktaufnahme erfolgte per E‑Mail oder telefonisch, dabei wurde über die Inhalte und Zielstellung der Befragung informiert und die Freiwilligkeit der Teilnahme betont. Bei schriftlicher Einwilligung zur Studienteilnahme wurde ein Termin zum Telefoninterview ausgemacht, welches nach Einholen des Einverständnisses aufgenommen, pseudonymisiert und vollständig transkribiert wurde.

Im Mai 2019 wurde unter dem Titel „Medizinische Versorgung von Asylsuchenden in Erstaufnahmeeinrichtungen“ eine eintägige, interaktive Fachtagung veranstaltet, zu welcher Teilnehmende der Interviewstudie sowie Vertreter*innen weiterer Standorte eingeladen wurden. Die Ergebnisse der vorangehenden Interviewstudie wurden den Teilnehmer*innen als Diskussionsgrundlage zurückgespiegelt. Die Tagung fand unter der Chatham House Rule (Regelung der Weitergabe von Inhalten vertraulicher Gespräche) statt, um einen offenen, aber dennoch vertraulichen Kommunikationsrahmen zu schaffen [12]. Alle Teile der Fachtagung wurden zur späteren Auswertung schriftlich dokumentiert; eine Kurzfassung der Dokumentation wurde den Teilnehmer*innen zur Verfügung gestellt.

**Figure 2:**
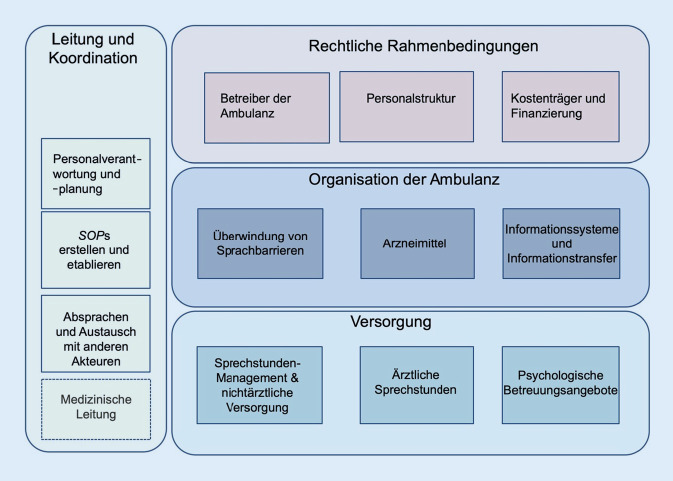

Für die Bestandsaufnahme (Ziel 1) erfolgte die Auswertung der Daten aus der Interviewstudie mittels qualitativer Inhaltsanalyse basierend auf der Framework-Methode [13]. Nach Transkription und mehrfachem Lesen der Interviews wurden basierend auf den WHO-Building-Blocks [14] Schlüsselthemen identifiziert, die wiederum in 4 Kategorien eingeteilt wurden (Abb. 2). Basierend auf diesem Schema ordneten sich die Teilnehmer*innen während der Tagung einer Kleingruppendiskussion zu. So entstanden 4 Kleingruppendiskussionen zu den Themen „Rahmenbedingungen: Betreiber, Kostenträger und Personal“, „medizinische und nichtmedizinische Versorgung“, „psychosoziale Versorgungsangebote“ und „Informationsaustausch und Dokumentationssysteme“. Daten wurden von der Erstautorin anhand dieses Schemas codiert und in einer Matrix organisiert, um die Daten der verschiedenen Teilnehmer*innen/Standorte zu vergleichen.

Für die Fragestellungen zu Herausforderungen und Lösungsansätzen (Ziele 2 und 3) wurde eine thematische Analyse [15] unter Einbezug der Interviews sowie der Dokumentation der Kleingruppen- und Podiumsdiskussion der Fachtagung durchgeführt. Die Codes wurden von der Erstautorin aus den Rohdaten induktiv erstellt und dann zu Subkategorien und Kategorien abstrahiert. Die Abstrahierung und Kategorisierung der Codes erfolgte gemeinsam mit den Moderator*innen der Fachtagung (LB, SN, RJ). Die unterschiedlichen Formen der Datenerhebung (Interviews/Kleingruppendiskussionen/Podiumsdiskussion) ermöglichten eine Triangulation der Daten. Iterativ war die Datenerhebung insofern, als dass die Ergebnisse der Interviews den Teilnehmern des Workshops rückgespiegelt wurden und von diesen diskutiert wurden; ebenso wurden basierend auf den Ergebnissen des Workshops neue Codes und Themen in die bereits erfolgte Analyse der Interviews eingearbeitet. Sofern nicht anders dargestellt, beziehen sich alle Ergebnisse auf die Daten nach Triangulation. Alle Analysen wurden mit MaxQDA (Version 12) durchgeführt. Die Studie erhielt am 22.06.2017 ein positives Votum der Ethik-Kommission der Medizinischen Fakultät Heidelberg (S-287/2017).

## Ergebnisse

Von 14 kontaktierten Personen nahmen 13 Personen aus 9 AE/zuständigen Landesbehörden in 4 Bundesländern an der Interviewstudie teil. An der Fachtagung nahmen 41 Personen aus 9 Bundesländern teil (Abb. 1).

### Qualitative Bestandsaufnahme der medizinischen Versorgung in Aufnahmeeinrichtungen in Deutschland

Die Interviewteilnehmer*innen berichteten, dass die teilnehmenden Ambulanzen von einer Vielzahl unterschiedlicher Akteur*innen betrieben wurden, darunter Universitätskliniken, der Öffentliche Gesundheitsdienst (ÖGD), die Kassenärztliche Vereinigung (KV) und private Betreiber, sowohl profitorientierte und als auch nichtprofitorientierte (Tab. 1). In den befragten Ambulanzen arbeiteten Ärzt*innen verschiedener Fachbereiche, Personal aus Gesundheitsfachberufen und Sozialarbeiter*innen (Tab. 1). Während private Betreiber fast ausschließlich mit Honorarärzt*innen arbeiteten, zeichneten sich die durch öffentliche Betreiber unterhaltenen Standorte durch eine Mischung aus angestelltem und honorarärztlich arbeitendem Personal aus.

**Table 1:** 

Komponente	Spektrum
**Rahmenbedingungen**	
Betrieb der Ambulanzen	Betreiber: – Öffentlich: Universitätskliniken, Kassenärztliche Vereinigung, Öffentlicher Gesundheitsdienst – Privat: profitorientiert und nichtprofitorientiert (z. B. kirchlich)
Personalstruktur	In den Ambulanzen arbeiteten: – Fachärzt*innen (an Uni auch Assistenzärzt*innen) – Meist zusätzlich verschiedene Angehörige von Gesundheitsfachberufen: Rettungssanitäter*innen, Notfallsanitäter*innen, Pflegekräfte und medizinische Fachangstelle (MFA) – An vereinzelten Standorten zusätzlich Sozialarbeiter*innen Arbeitsverhältnis in Teilen abhängig vom Betreiber: – Private Betreiber: Vor allem Honorarärzt*innen – Öffentliche Betreiber: Mischung aus Honorarärzt*innen und Angestellten (Bruttostundenlohn für Honorarärzt*innen variierte zwischen 75 € und 130 €, häufig waren diese bereits im Ruhestand)
Praktische Auswirkungen des AsylbLG auf die medizinische Versorgung	An einem Standort: Definition des Leistungskatalogs durch Gesundheitskarte An allen anderen Standorten: Antrag auf Kostenübernahme notwendig für – Sehr teure Behandlungen oder Medikamente – Alle Medikamente Vom Ambulanzpersonal wahrgenommenes Spektrum der Versorgung: – Stark eingeschränkt bis – Sehr nah an Regelversorgung
**Versorgung**	
Ärztliche Versorgung	Angebotene Fachrichtungen: – Immer: Allgemeinmedizin (2,6–10 h/100 Bewohner*innen) – z. T. zusätzliche Fachrichtungen, u. a. Gynäkologie, Pädiatrie, Zahnmedizin, Dermatologie
Psychosoziale Versorgung	Häufig keinerlei psychosoziale Versorgung, wenn Versorgung angeboten, dann: – „Stabilisierungssprechstunde“ – Psychotherapeutische Sprechstunde in der Ambulanz – Anbindung an eine psychiatrische Ambulanz der Uniklinik – „Psychosoziale“ Sprechstunde (Dennoch berichteten alle Standorte, den psychologischen Bedarf der Bewohner*innen der AE nicht ausreichend adressieren zu können.)
Sprechstundenmanagement und nichtärztliche Versorgung	Große Unterschiede in den Aufgaben des Gesundheitspersonals: – Terminorganisation mit externen Versorgern – Durchführung von EKG, Labor – Durchführung von „pflegerischen“ Tätigkeiten, wie Wundversorgung, Triage und Erstversorgung – Dokumentation und Assistenz in den Sprechstunden – Individuelle Betreuung und Nachverfolgung der einzelnen Patient*innen – „Pflegerische Sprechstunde“ (einfache medizinische Versorgung bei Erkältung, Magen-Darm-Beschwerden etc.) – Organisation des Medikamenten- und Hilfsmittelbestands – Erstellung von Dienstplänen – Betreuung und Organisation weiteren Personals (Dolmetscher/Security)
**Organisation und Leitung**	
Sprachmittlung	Unterschiede in der Finanzierung von Sprachmittler*innen: – Keine Finanzierung, nur Familienmitglieder und Freunde als Sprachmittler*innen – Bezahlte Dolmetscher*innen verfügbar bei bestimmten Indikationen (z. B. Aufklärung zu Operationen, psychologische Versorgung, Überweisung in die Regelversorgung) – Immer Dolmetscher*innen vor Ort für häufigste Sprachen Unterschiedliche Konzepte: – „Laiensprachmittler*innen“, z. T. auch Medizinstudierende – Vereidigte Dolmetscher*innen – Videodolmetschsysteme für ärztliches Personal und Leitstelle
Ausgabe von Rezepten und Arzneimitteln	Mögliche Prozesse: – Medikamentenstandard vor Ort (z. T. sehr umfangreich, z. T. nur das Allernötigste) – Ausgabe von Rezepten; diese wurden von Asylsuchenden in einer Apotheke der Wahl eingelöst – Lieferung nach Ausstellung des Rezeptes von städtischen Apotheken
Dokumentations- und Informationssysteme	Dokumentation der Versorgung entweder – Papierbasiert (vor allem am Anfang) – Elektronische Dokumentation; gebräuchliche Praxissysteme: – Spezifische Software des Betreibers für die gesamte Unterkunft – Klinikinformationssysteme (KIS) – Refugee Care Manager (RefCare©)

Alle Interviewteilnehmer*innen berichteten über eine allgemeinmedizinische Sprechstunde und je nach Standort zum Teil zusätzliche Fachrichtungen, darunter Gynäkologie, Pädiatrie, Zahnmedizin und/oder Dermatologie. Ein psychologisches Versorgungsangebot wurde vereinzelt angeboten.

Für das Gesundheitsfachpersonal wurde ein sehr vielseitiges und standortabhängig auch ein sehr unterschiedliches Aufgabenspektrum beschrieben. Dazu gehörten Aufgaben wie Sprechstundenmanagement und Terminvergabe, aber z. T. auch pflegerische Aufgaben und Notfallversorgung. Außerdem wurde berichtet, dass auch organisatorische Treffen mit anderen Akteur*innen, etwa dem Kostenträger, Sozialdiensten und Honorarärzt*innen, häufig zum Aufgabenspektrum dieser Berufsgruppe gehörten. Dem nichtärztlichen Gesundheitspersonal kam eine Schlüsselposition bei der Informationsweitergabe innerhalb der Ambulanz zu („Ich glaub’ die Brückenperson bin halt ich vor Ort, weil ich arbeite mit allen Disziplinen zusammen“, Person 13, Ort 9 [Versorger*in]).

Die meisten Interviewteilnehmer*innen mussten für Arzneimittelverordnungen oder weiterführende externe Behandlungen Anträge auf Kostenübernahme an den Kostenträger stellen, damit dieser über die Konformität der Behandlung gemäß AsylbLG entscheiden konnte. Nur an einem Standort war durch die Einführung der Gesundheitskarte der Leistungskatalog klar definiert. Große Unterschiede zeigten sich hinsichtlich der zu bewilligenden und abzulehnenden Maßnahmen: An einigen Standorten musste nur für alles, „was sehr, sehr kostenintensiv ist“ (Interview 2, Ort 2 [Behörde]), ein Antrag gestellt werden, während an einem anderen Standort jede einzelne Arzneimittelverordnung oder -ausgabe vom Kostenträger bewilligt werden musste. Auch das Ausmaß der tatsächlichen Versorgung variierte: An einigen Standorten wurde eine Versorgung beschrieben, die sich nach medizinischem Ermessen richtete und „fast nicht eingeschränkt“ (Person 9, Ort 4 [Versorger*in]) sei, während woanders „massive Probleme mit Nichtbewilligung“ notwendiger Leistungen (Person 4, Ort 3 [Versorger*in]) beschrieben wurden.

Eine ausführliche Zusammenstellung der Versorgungskonzepte befindet sich in Tab. 1.

### Herausforderungen beim Aufbau nachhaltiger und bedarfsgerechter Versorgungstrukturen

#### Im Spannungsfeld zwischen lokalen, kontextabhängigen Lösungen und einheitlichen Vorgaben

Die Heterogenität der beschriebenen Strukturen (Tab. 1) lässt sich zu einem großen Teil auf die sehr unterschiedlichen Voraussetzungen an den einzelnen Standorten zurückführen. Insbesondere das Vorhandensein und die Initiative lokal aktiver Akteur*innen, wie dem ÖGD, niedergelassenen Ärzt*innen und Universitätskliniken, prägten die Versorgungsstrukturen entscheidend. Ähnlich den unterschiedlichen Kontextfaktoren (Großstadt/Kleinstadt/ländlicher Raum) bestimmt die Verfügbarkeit der medizinischen Versorgungsstrukturen, etwa der psychosozialen Versorgung, der möglichen Sprachmittler*innen, der ehrenamtlichen Helfer*innen oder der suchtmedizinischen Versorgung, nicht zuletzt auch die Umsetzbarkeit verschiedener Versorgungskonzepte.

Die Gewährung eines sinnvollen Handlungs- und Interpretationsspielraums bei gleichzeitiger Etablierung landesweiter Versorgungskonzepte wurde als Herausforderung für die zuständigen Landesbehörden identifiziert. Die Heterogenität bereitet in vielen Bereichen der Versorgung Probleme, etwa bei der Finanzierung von Sprachmittlung, psychosozialer Versorgung oder Lösungen zum Informationsaustausch.

#### Formalisierung der Arbeitsprozesse in der Ambulanz

Nicht nur die Etablierung landesweiter Versorgungskonzepte, sondern auch die Formalisierung von Arbeitsprozessen innerhalb der Ambulanz, welche 2015 zunächst als schnelle Reaktion auf die zunehmende Anzahl an Asylsuchenden stattfanden, stellt weiterhin eine große Herausforderung dar: „Wir sind noch im Notfallmodus“ (Person 3; Podiumsdiskussion).

Der Wunsch nach der Erstellung von Standardabläufen zog sich durch alle Komponenten der medizinischen Versorgung und betraf vor allem die Erstellung und Bewilligung von Anträgen auf Kostenübernahme, die Bestellung und Ausgabe von Medikamenten und vorgehaltene Medikamentenstandards, den datenschutzkonformen Empfang und die Weitergabe von patientenbezogener Information, den Umgang mit psychologisch/psychiatrisch auffälligen Bewohner*innen, inkl. Suchterkrankungen, und die Notfallversorgung außerhalb der Sprechzeiten. Außerdem wurden die Zusammenarbeit und der Informationsaustausch zwischen den vielen Akteur*innen der AE als problematisch beschrieben. Auch die mangelnde Zuordnung von Aufgaben zu bestimmten Personen oder Positionen in der Ambulanz führte dazu, dass anfallende Strukturierungsprozesse und organisatorische Aufgaben von verschiedenen Akteur*innen übernommen und auch in unterschiedlichem Ausmaß adressiert wurden.

In den Interviews mit den Versorger*innen wurden verschiedene für die Formalisierung der Versorgung hinderliche Faktoren beschrieben. Ein mehrfach genanntes Problem war der häufige Wechsel der Betreiber, der durch die regelmäßigen Ausschreibungen bedingt war. Auch die Notwendigkeit einer sehr flexiblen Planung, d. h. die Möglichkeit, Personal ohne lange Vorlaufzeit einsetzen oder reduzieren zu können, wurde als hinderlich beschrieben. Bedingt war dies durch die stark schwankenden Zahlen von Asylsuchenden und die häufige Umstrukturierung von Erstaufnahmeeinrichtungen. Häufig wurde Unsicherheit bezüglich des Fortbestands der einzelnen Einrichtungen genannt:

Wir [haben] ganz klar gesagt …, wir haben hier wirklich ein kleines Kompetenzzentrum für Menschen auf der Flucht. Weil wir machen ja auch Beratung, wir machen ja auch psychologische Betreuung. Und das wird alles abgebaut, dann muss man’s woanders wieder aufbauen (Person 8, Ort 2).

Bedingt durch die Notwendigkeit einer flexiblen und unverbindlichen Planung wurden an vielen Standorten Honorarkräfte eingesetzt. Das daraus resultierende Arbeitsverständnis einer „medizinischen Dienstleistung“ und die generell mangelnde Zuständigkeit wurden als hinderlich für den Formalisierungsprozess identifiziert.

#### Belastung des Personals

Interviewteilnehmer*innen beschrieben, dass die Arbeit in AE auch auf persönlicher und emotionaler Ebene sehr herausfordernd sei. Dazu gehören zum einen Herausforderungen wie unklare Aufgabenbereiche, Arbeitsabläufe und Kompetenzen, aber auch das politische und rechtliche Umfeld. Sehr häufig wurde eine unklare gesetzliche Lage als belastend beschrieben, darunter vor allem die Arbeit unter dem AsylbLG. Das Ambulanzpersonal beschrieb sich dabei in einer Vermittlerposition zwischen dem Kostenträger und den Asylsuchenden, war beim Kostenträger Fürsprecher für die medizinischen Bedarfe der Asylsuchenden und musste gleichzeitig den Asylsuchenden negative Bescheide erklären. Auch in anderen Bereichen empfanden sie sich als Mittler*innen zwischen Konfliktparteien, etwa zwischen Bewohner*innen der AE und der Ortsgemeinde oder zwischen Kostenträger und Betreiber mit unterschiedlichen finanziellen Interessen. Als belastend wurde zudem die Arbeit mit „pragmatischen“ Lösungen beschrieben, die nach dem Verständnis der Arbeitenden keine optimale Versorgung ermöglichten. Dies betraf vor allem ungedeckte psychologische Bedarfe sowie die Arbeit mit Sprachbarrieren.

### Lösungsvorschläge und Handlungsbedarfe

Die vielfachen Herausforderungen der medizinischen Versorgung in AE müssen koordiniert und strukturiert adressiert werden: „Es gibt viel aufzuräumen“ (Podiumsdiskussion, Person 3).

Als gesetzlich Verantwortliche und Kostenträger für die Versorgung der Asylsuchenden in AE wurden die *zuständigen Landesbehörden* als wichtige Akteure identifiziert; diese Ansicht wurde auch von anwesenden Vertreter*innen von Landesbehörden geteilt. Bezüglich des beschriebenen Spannungsfeldes zwischen lokalen Lösungen und einheitlichen Standards wurde sich generell für die Beibehaltung der unterschiedlichen Versorgungsmodelle (d. h. z. B. Art des Betreibers, Arbeitsverhältnis des Personals) ausgesprochen, um individuellen Bedarfen und Herausforderungen der unterschiedlichen Standorte gerecht werden zu können. Für einige spezifische Bereiche wurde jedoch explizit die Notwendigkeit zentraler Vorgaben und Standards vonseiten der Landesbehörde herausgearbeitet, entweder in Form von einrichtungsübergreifenden Versorgungskonzepten oder in der umfassenderen Ausgestaltung der Verträge mit den einzelnen Betreibern der Ambulanzen (Tab. 2). So sollten organisatorische Aufgaben und Leitungsfunktionen benannt, zugewiesen und vergütet werden und die Erstellung und Etablierung von Standardabläufen, regelmäßigen Vernetzungstreffen und die Koordination relevanter Akteur*innen genauso Teil des Versorgungsauftrags sein wie das Angebot medizinischer Sprechstunden. Auch wurde die Ausgestaltung eines Leistungskatalogs, wie er z. B. in Sachsen existiert, diskutiert. Dieser könne die Nachvollziehbarkeit der Entscheidungen für Asylsuchende verbessern und die Kontinuität der Einzelentscheidungen erhöhen. Weitere Bereiche, in denen zentrale Vorgaben notwendig seien, wären die Etablierung eines einrichtungsübergreifenden, datenschutzkonformen Dokumentationssystems und die Erarbeitung und Finanzierung eines Konzeptes zur Sprachmittlung.

**Table 2:** 

Akteure	Erarbeitete Lösungsstrategien und Handlungsbedarf
Landesbehörde	*Vertragliche Ausgestaltung mit Betreibern:* – Definition und Vergütung von Organisations- und Leitungsfunktionen – Vorgaben zur Erstellung und Etablierung von Standardabläufen und Vernetzungstreffen – Koordination relevanter Akteur*innen innerhalb des Versorgungsauftrags – Kontinuität in der Versorgung sicherstellen: Nicht viele unterschiedliche Versorger*innen mit wenigen Stellen und Wechsel in der Personalbesetzung vermeiden Sprachmittlung: Ausarbeitung und Finanzierung von Konzepten zur Sicherstellung der für die medizinische Versorgung notwendigen Kommunikation Dokumentation und Informationsweitergabe: – Schnittstellen zwischen den einzelnen Einrichtungen (Ambulanzen untereinander, aber auch Öffentlicher Gesundheitsdienst) technisch und datenschutzkonform ermöglichen
Öffentlicher Gesundheitsdienst	Psychosoziale Versorgung: – Vermehrt präventive Konzepte für die psychosoziale Versorgung in die Formulierung einbringen Schnittstellen zu kurativer Versorgung aufbauen: – Kommunikationskanäle etablieren, um identifizierte Vulnerabilitäten und z. B. Impfbedarfe effizienter mit kurativen Versorgern auszutauschen Zentrale Steuerung und Qualitätsmanagement: – Diskussion der Einbringung des Öffentlichen Gesundheitsdienstes in Leitung und Organisation der Ambulanzen
Wissenschaft	Transfer von Wissenschaft zu Praxis erleichtern: – wissenschaftliche Berichte in Form von kurzen Berichten verfassen, Kommunikation an Politik verstärken Forschungsbedarfe adressieren: – Interventionsstudien zu unterschiedlichen Versorgungsmodellen, Triage und Notfallversorgung, Kosteneffektivität von Dolmetschern
Ambulanzpersonal	Fort‑/Weiterbildung des Ambulanzpersonals: – Schulung im Umgang mit psychosozialen Problemstellungen, integrierten Versorgungskonzepten und Fähigkeiten des Case-Managements Erfahrungsaustausch: – Vernetzung mit anderen Ambulanzen und anderen Akteur*innen auf dem Gelände der Aufnahmeeinrichtung stärker ausbauen Bedarfe erfassen: – Aktiv Schwankungen in der Inanspruchnahme von Versorgung wahrnehmen und Unter‑/Überangebot der angebotenen Sprechstunden wahrnehmen und kommunizieren

Es wurde außerdem die Rolle der *lokalen Gesundheitsämter *diskutiert, auch von deren anwesenden Vertreter*innen. In mehreren Komponenten der medizinischen Versorgung wurde sich eine verstärkte Einbindung in Form einer leitenden und verknüpfenden Instanz des ÖGD gewünscht. Der ÖGD müsse seine „Scheuklappen entfernen“ (Kleingruppendiskussion Versorgung), d. h. seinen Fokus von Infektionskrankheiten erweitern und sich im Rahmen des Präventionsgesetzes auch z. B. um präventive Aspekte der psychosozialen Versorgung, die verstärkte Vernetzungen von Gesundheitsuntersuchungen, Impfbedarfen und die Verzahnung präventiver und kurativer Versorgung kümmern. Auch eine zentrale Steuerung und ein mögliches Qualitätsmanagement durch den ÖGD wurden diskutiert.

Auch für die *Wissenschaft* wurden Anregungen gegeben: Um den Transfer wissenschaftlicher Erkenntnisse an die Praxis zu erleichtern, wurden sich kurze Berichte gewünscht, welche „die Wissenschaft verständlich machen für Behörden“ (Person 1, Podiumsdiskussion). Auch Forschungsbedarfe wurden aufgezeigt, wie Interventionsstudien zu den unterschiedlichen Versorgungsmodellen und sinnvolle Strategien zur Triage und Notfallversorgung in AE.

Eine weitere, wichtige Ebene stellt die *Ambulanz* selber dar; auch hier wurden direkte Empfehlungen erarbeitet. Das an vielen Standorten informell etablierte Case-Management, d. h. die individuelle organisatorische Betreuung und Nachverfolgung von Patient*innen, sollte ausgebaut und formalisiert werden; dazu sei auch die Fort‑/Weiterbildung des Ambulanzpersonals notwendig. Ungedeckte Bedarfe, auch eine Unter‑/Überlastung der Ambulanzkapazitäten müssten aktiv wahrgenommen und erhoben werden, um zeitnah an Kostenträger kommuniziert zu werden.

*Bundesweite* Lösungen wurden zur Erleichterung der Integration von Asylsuchenden in die Regelversorgung gefordert: Nötig sei die Finanzierung von Sprachmittlung, auch in der Regelversorgung, und die Vergütung des zeitlichen Mehraufwands in der ärztlichen Versorgung.

## Diskussion

Unsere kombinierte Datenerhebung aus Einzelinterviews, Kleingruppen- und Podiumsdiskussion mit Teilnehmer*innen aus mehreren Bundesländern Deutschlands stellt umfassend die Heterogenität der seit 2015 entstandenen medizinischen Versorgungsstruktur in Aufnahmeeinrichtungen für Asylsuchende dar.

Insgesamt zeichneten sich die untersuchten Versorgungskonzepte vor allem durch ihre Varianz aus. Diese Varianz führt dazu, dass die Qualität und das Ausmaß der Versorgung standortbedingt unterschiedlich sind: Ob eine Behandlung unter dem AsylbLG als zulässig erklärt wird, ob eine psychosoziale Versorgung angeboten wird und ob die Kommunikation mit dem medizinischen Personal möglich ist, hängt von der Zuteilung der Asylsuchenden zu den einzelnen AE ab. Um nicht eine „Lotterie“ der gesundheitlichen Ungleichbehandlung entstehen zu lassen, müssten bundesweite Mindeststandards der medizinischen Versorgung definiert werden. Deren Umsetzung sollte durch lokale Akteur*innen in der vertraglichen Ausgestaltung mit den regional spezifischen Kostenträgern gewährleistet werden, besonders an Standorten mit häufigem Betreiberwechsel. Idealerweise müsste diese auch eine Erhebung von und Anpassung an lokale Bedarfe beinhalten.

Bisher hat es jedoch an mehreren Standorten einen Mangel an Vorgaben vonseiten der Kostenträger bezüglich der Ausgestaltung der medizinischen Versorgung gegeben. Dies hat dazu geführt, dass die Versorgungskonzepte „von unten“ und bedingt durch die komplexen Herausforderungen vor Ort entworfen wurden. Obwohl die Möglichkeit, lokal flexibel zu agieren, in einigen Bereichen als durchaus positiv bewertet wurde, fehlte es vor allem an einer Vernetzungs- und Koordinationsinstanz, durch die im gemeinsamen Austausch mit Kostenträgern und anderen Ambulanzen Mindeststandards zur Qualitätssicherung hätten definiert werden können. An einigen Standorten kam hinzu, dass durch die Anstellung von Ärzt*innen als Honorarkräfte auch eine ärztliche Leitung in der Ausgestaltung des Versorgungskonzeptes ausblieb. Die Leitungs- und Koordinationsfunktion fiel in diesen Fällen auf das nichtärztliche Gesundheitsfachpersonal, welches nun eine Doppelrolle (Koordination und Gesundheitsversorgung) einnahm. Unsere Studie zeigt, dass diese Rollenverteilung, und vor allem deren impliziter Charakter, zu einer starken physischen und psychischen Belastung des Personals führt. Um Kontinuität in der Versorgung zu gewährleisten und einen häufigen Personalwechsel zu vermeiden sowie ein akzeptables Arbeitsumfeld für das Personal zu schaffen, ist es essenziell, die Leitung „von oben“ – von behördlicher als auch ärztlicher Seite – zu stärken. Zudem sollte in Erwägung gezogen werden, die implizit entstandene Koordinationsfunktion, welche für den Betrieb der Ambulanz und der interdisziplinären Vernetzung mit verschiedenen Akteur*innen essenziell ist, weiter zu formalisieren. Aus anderen Ländern gibt es bereits positive Erfahrungswerte mit der Berufsgruppe Refugee Health Nurse [16], aus denen für die Institutionalisierung dieser Koordinationsfunktion gelernt werden könnte.

Ein weiterer Bereich, in dem das „Leitungsvakuum“ in der medizinischen Versorgung von Geflüchteten deutlich wurde, ist die Ausgestaltung des Asylbewerberleistungsgesetzes. Durch die vage Formulierung des Gesetzes und fehlende Richtlinien zu dessen Interpretation verschieben sich die Konsequenzen auf Versorgungsebene: Das Gesundheitsfachpersonal versteht sich als „Lückenfüller“ zwischen den Bedarfen der Patient*innen und den unklaren Vorgaben des Kostenträgers und übernimmt somit zusätzlich zur medizinischen Versorgung implizit die Aufgabe, die Erwartungen und Anforderungen beider Seiten in Einklang zu bringen und auszuhandeln.

In Bezug auf die identifizierten Herausforderungen und Handlungsbedarfe zeigt unsere Bestandsaufnahme große Parallelen zu einer 2015 durchgeführten bundesweiten Situationsanalyse der medizinischen Versorgung Geflüchteter [11]. Die dort beschriebenen Herausforderungen weisen Ähnlichkeiten auf, besonders hinsichtlich der institutsübergreifenden, standardisierten Kommunikation von identifizierten medizinischen Bedarfen und Vulnerabilitäten, aber auch hinsichtlich der Konzepte zur Sprachmittlung und der gezielten Fort- und Weiterbildung von Personal. Insofern sind Vorschläge zur Vereinheitlichung und Standardisierung verschiedener Bereiche wie Unterbringung [17], Erstuntersuchung [18, 19], psychosoziale Versorgung [20], Zugang zur Versorgung und Umgang mit der elektronischen Gesundheitskarte (eGK; [21]) weiterhin von besonderer Relevanz.

Um die Reaktionsfähigkeit eines Gesundheitssystems auf akute oder chronische Veränderungen einzuschätzen, schlagen Blanchet et al. [22] das Konzept der „Gesundheitssystem-Resilienz“ vor. Demnach sind, je nach der Schwere und Reichweite der Veränderungen, die absorptiven, adaptiven und/oder transformativen Fähigkeiten des Systems gefragt. Bereits 2015 zeigte sich, dass eine Reaktion auf die steigende Anzahl an Flüchtlingen im rein absorptiven Sinne nicht ausreicht: Bestehende gesundheitliche Versorgungsangebote müssen z. B. im Sinne der Sprachmittlung, des Umgangs mit psychischen Belastungen, aber auch hinsichtlich der Kostenabrechnung über eine elektronische Gesundheitskarte angepasst werden. Allein die Einrichtung von medizinischen Ambulanzen in den AE zeigte zudem, dass auch die transformative Kapazität des Systems gefragt war [23]. Jedoch wurden einzelne Initiativen wie die Sprachmittlung per Videodolmetscher*in [6, 7], die niederschwellige Versorgung von psychischen Belastungen [24], die Einrichtung von elektronischen Dokumentationssystemen [25] oder die Kostenabrechnung per elektronischer Gesundheitskarte [26] noch nicht systematisch implementiert. Unsere Erhebung zeigt, dass das deutsche Gesundheitssystem seine transformative Reaktionsfähigkeit auf Fluchtmigration auch im Bereich der Koordination der Ambulanzen, der Auslegung des Asylbewerberleistungsgesetzes und der Erstellung von bundesweiten Mindeststandards für Ambulanzen in AE beweisen muss, um den weiterhin bestehenden Problemen in der Bereitstellung bedarfsorientierter Versorgung entgegenzuwirken. Trotz sinkender Asylantragszahlen bedarf es eines stetigen Engagements in der Verbesserung der gesundheitlichen Lage für Geflüchtete, wobei die Leitung durch Behörden auf Bundes- und Länderebene besonders gefragt ist.

### Stärken und Limitationen der Studie

Dies ist die erste Studie, die die Barrieren einer bedarfsgerechten Versorgung in den medizinischen Ambulanzen der AE für Geflüchtete in Deutschland regionenübergreifend systematisch darlegt. Die Stärken dieser Studie liegen in dem gewählten iterativen und partizipativen Ansatz zur Datenerhebung. Durch langjährige praktische und wissenschaftliche Erfahrung der Autor*innen in der gesundheitlichen Versorgung von Geflüchteten in Ambulanzen von AE konnte für die Einzelgespräche ein Leitfaden entwickelt werden, welcher auf wesentliche Dimensionen der Versorgung vor Ort einging. Im Rahmen des partizipativen Workshops konnten identifizierte Barrieren mit relevanten Akteur*innen vergleichend vertieft und Lösungsvorschläge gemeinsam erarbeitet werden. Zudem war an der Analyse der qualitativen Daten ein interdisziplinäres Team von Wissenschaftler*innen mit ärztlichem und nichtärztlichem professionellen Hintergrund, inklusive Vertreter*innen aus dem Ambulanzbetrieb, dem Öffentlichen Gesundheitsdienst und der Wissenschaft vertreten.

Die Studie ist jedoch eingeschränkt in ihrem exklusiven Fokus auf die Barrieren der Versorgung in AE, sodass spezifische Aspekte der gesundheitlichen Versorgung von Geflüchteten auf Ebene der Stadt- und Landkreise sowie der Kommunen nicht unmittelbar beleuchtet wurden. Die Versorgungssituation aus der Perspektive von Asylsuchenden wurde hier nicht berücksichtigt, ist jedoch in anderen Datenerhebungen des RESPOND-Projekts erfolgt und wird die hier berichteten Perspektiven ergänzen. Der Einbezug von Studienteilnehmer*innen aus allen 16 Bundesländern statt aus 4 (in Interviews) bzw. 9 (in den Workshops) hätte möglicherweise die „Generalisierbarkeit“ der Erkenntnisse erhöht. Gleichzeitig können Bundesländer angesichts des qualitativen Ansatzes der Forschung die Transferabilität der Forschungsergebnisse in ihren jeweiligen Kontext überprüfen.

## Fazit

Die Studie zeigt den organisatorischen, rechtlichen und administrativen Handlungsbedarf in AE auf, der auch 4 Jahre nach der Einwanderung einer hohen Zahl von Schutzsuchenden nach Deutschland besteht. Obgleich die akuten Herausforderungen der Sicherstellung der Gesundheitsversorgung für Geflüchtete bewältigt wurden, ist die mittel- und langfristige Ausbildung von Strukturen und Prozessen teilweise ausgeblieben. Aus der Perspektive der Organisations- und Systementwicklung ist Führungsarbeit gefragt, die – jenseits föderaler Zuständigkeiten – die gewachsenen Versorgungsstrukturen rahmengebend durch Standards auf ein nachhaltiges und zukunftsfähiges Fundament stellt. Angesichts der zunehmenden Aufenthaltsdauer in den AE ist es normativ geboten, vergleichbare Bedingungen der gesundheitlichen Versorgung von Geflüchteten im Bundesgebiet zu schaffen. Die durch das partizipative Forschungsvorhaben angestoßenen Denk- und Diskussionsprozesse sollten durch regionenübergreifende Netzwerkbildung aus Behörden, ärztlichen Akteur*innen, ÖGD und Wissenschaft im Sinne einer Joint-Learning-Initiative fortgeführt und institutionalisiert werden.
